# Comparative Analysis of the Physical Properties of Three Different Tricalcium Silicate Cements: An *In Vitro* Study

**DOI:** 10.1055/s-0045-1809311

**Published:** 2025-05-29

**Authors:** Nandini Devi M., Ganesh Jeevanandan, Prabhadevi C. Maganur, Hammam Ahmed Bahammam, Suman Panda, Ather Ahmed Syed, Satish Vishwanathaiah

**Affiliations:** 1 Department of Pedodontics and Preventive Dentistry, Saveetha Dental College and Hospitals, Saveetha Institute of Medical and Technical Sciences, Saveetha University, Chennai, Tamil Nadu, India; 2 Division of Pediatric Dentistry, Department of Preventive Dental Sciences, College of Dentistry, Jazan University, Jazan, Saudi Arabia; 3 Department of Pediatric Dentistry, Faculty of Dentistry, King Abdulaziz University, Jeddah, Saudi Arabia

**Keywords:** biodentine, mineral trioxide aggregate (MTA), compressive strength, dental materials

## Abstract

**Objective:**

Calcium trisilicate materials, including biodentine, are popular choices for dental restorations owing to their biocompatibility. However, their compressive strength often falls short of ideal levels for certain restorative procedures. This research investigates the new biodentine formulation in the market, augmented with barium oxide to improve compressive strength and examines its physical characteristics.

**Materials and Methods:**

Three distinct material groups were subjected to compressive strength testing. Group A comprised mineral trioxide aggregate (white MTA, Angelus, Londrina, Brazil), group B consisted of biodentine (Septodont, Saint-Maur-des-Fossés, France), and group C featured the new commercially available biodentine formulation in the market, Kedo Bio D+ (Kedo Dental, Chennai, India). A universal testing machine was employed to conduct the compressive strength tests under standardized conditions.

**Results:**

Across all time intervals, group C consistently displayed the highest average compressive strength, followed by group B, while group A showed the lowest values. The differences in compressive strength between the three groups were statistically significant ( *p* = 0.001) after 7 days. The new biodentine formulation in group C withstood the highest maximum force, approximately 1,639 N, emphasizing its superior compressive strength compared with the other materials.

**Conclusion:**

This study demonstrated that the incorporation of barium oxide in Bio D+ formulation significantly enhanced its compressive strength compared with conventional biodentine and MTA. Kedo Bio D+ exhibited superior mechanical properties, making it a promising material for dental restorations requiring increased strength and durability.

## Introduction

Biodentine, a material based on tricalcium silicate, has transformed restorative and regenerative dentistry by providing a biologically favorable and versatile alternative to traditional materials like mineral trioxide aggregate (MTA). 1 2 Marketed as “dentin in a capsule,” biodentine is free from impurities commonly associated with Portland cement derivatives. 3 Its composition includes tri- and dicalcium silicates, calcium carbonate, zirconium dioxide, and a liquid phase containing calcium chloride in an aqueous solution. 3 This unique composition endows biodentine with exceptional biocompatibility, ease of use, low solubility, rapid setting time, and high compressive strength. 3 Its ability to stimulate mineralization, promote reparative dentin formation, and provide effective sealing makes it suitable for a wide range of dental applications. 4 Biodentine is particularly effective in vital pulp therapies, such as direct and indirect pulp capping, and serves as a reliable liner beneath composite restorations. 5 6

Biodentine's adaptability extends well beyond its traditional restorative roles. It functions as a substitute for dentin in posterior restorations, an alternative to enamel in minimally invasive caries management, and as a protective cavity liner for the pulp in cases of deep caries. 7 In pediatric dentistry, biodentine is especially useful for indirect pulp treatment, a conservative method aimed at managing deep caries while maintaining pulp vitality, particularly in children who may not cooperate with complex procedures. 8 9 10 This method reduces the likelihood of pulp exposure and avoids the risks associated with more invasive treatments. 11 Furthermore, biodentine's ability to be applied in bulk without requiring additional surface preparation simplifies its use, making it a practical option in demanding clinical situations. 12

Biodentine, while highly advantageous, does have some drawbacks. Porosity and the risk of microleakage at the junction between the material and dentin have been observed. 13 14 These issues often stem from incomplete hydration of cement particles and improper mixing ratios of water to cement, creating small voids that reduce the material's mechanical strength. 13 Enhancing its formulation is vital to overcoming these limitations and improving its performance in clinical applications.

Biodentine's mechanical characteristics, particularly its compressive strength, are fundamental to its success as a restorative material. Its strength allows it to withstand occlusal forces and provide structural stability, similar to natural dentin, making it effective for load-bearing restorations. 15 Acknowledging the need for mechanical durability in clinical use, this study introduces an advanced biodentine formulation designed to enhance compressive strength. By optimizing its components, the study aims to improve the material's robustness and extend its potential applications in restorative dentistry.

## Materials and Methods

This *in vitro* study was performed at the scientific materials research facility. The study's experimental design, procedures, and material composition were reviewed and authorized by the institutional human ethical committee [IHEC /SDC/PEDO-2103/23/128-A].

### Preparation of Test Materials

**Fig. 1:**
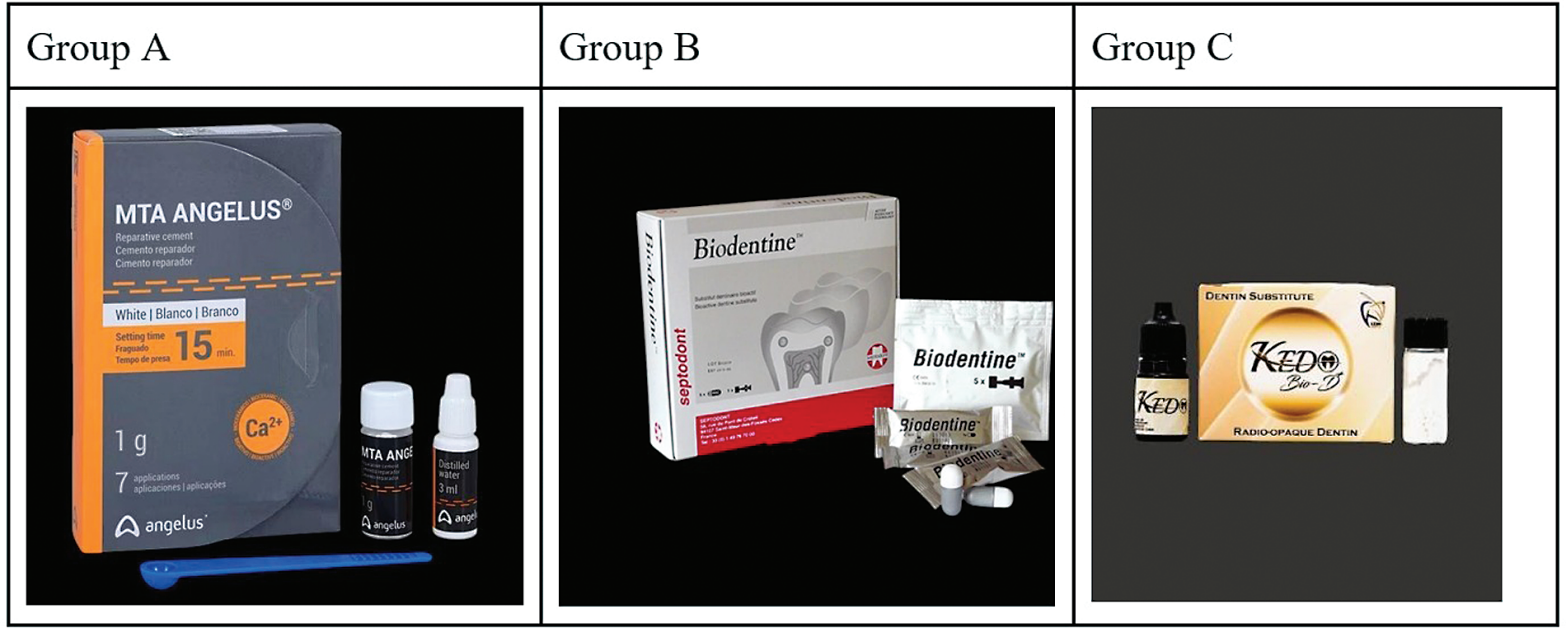
Materials used in the current study.

Two commercially available bioactive bioceramics and a newly modified biodentine were used in the present study. The commercially available materials used in the present study ( Fig. 1 ) were:

Group A: MTA, sourced from Angelus (Londrina PR, Brazil), is provided in a powder-liquid form. In accordance with the manufacturer's instructions, a 3:1 ratio of powder to liquid was measured and placed on a mixing pad. The powder was fully incorporated with the liquid until the mixture reached a thick consistency. The prepared material was then transferred using an MTA carrier to the specified experimental design.

Group B: Biodentine, supplied by Septodont (Saint Maur des Fossés, France), is provided in a powder-liquid form. As per the manufacturer's guidelines, the liquid was added dropwise to the powder in the capsule and mixed using a mechanical triturator from Dentsply Maillefer (Tulsa, United States) for approximately 30 seconds.

Group C: This group included the biodentine recently sold in the market, Kedo Bio D+ in the current study, which featured an updated formulation containing tricalcium silicate (70–72%), dicalcium silicate (6–8%), calcium carbonate (12–16%), calcium sulfate (1–3%), and barium oxide (2–4%) as the primary powder components. The core materials, tricalcium silicate and dicalcium silicate, were synthesized in the laboratory following the method described by Moon et al. 16 Calcium chloride, obtained in powder form from Tokyo Chemical Industry India Pvt. Ltd. (Chennai, India), was combined with 1 mL of distilled water to prepare a 30% concentration, which served as the liquid component. A 3:1 powder-to-liquid ratio was measured and placed on a mixing pad. The powder was fully hydrated with the liquid, and the mixing continued until a uniform, moldable putty-like consistency was achieved, allowing the material to be manipulated into a ball shape for easier handling. The final mixture was then transferred to the experimental design using a plastic instrument.

**Fig. 2:**
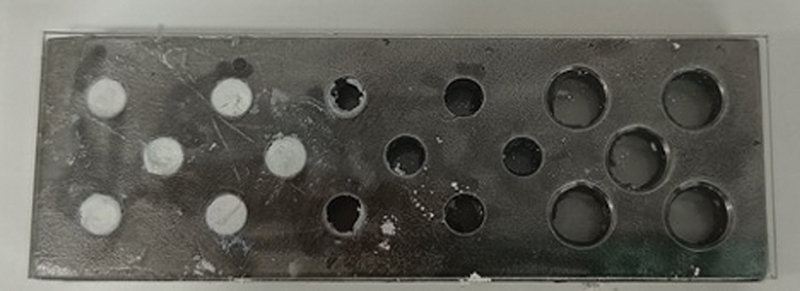
Stainless steel molds packed with samples and covered with glass slides.

**Fig. 3:**
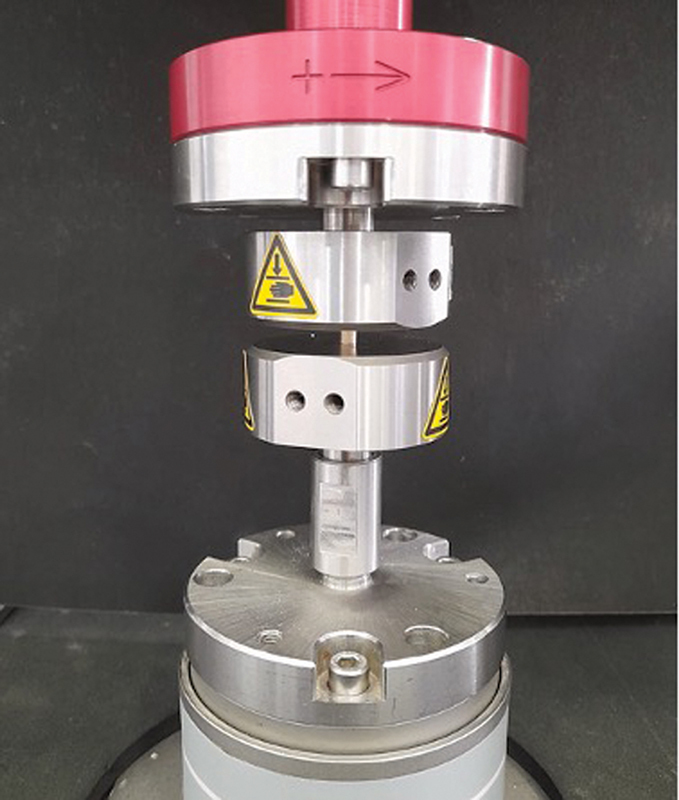
Compressive strength testing of material samples using the Instron Universal Testing Machine.

To evaluate the compressive strength of the experimental materials, the procedure outlined in American National Standards Institute/American Dental Association No. 96 was followed, utilizing stainless steel molds with a diameter of 4 mm and height of 6 mm. The laboratory environment was maintained at a controlled temperature of 23 ± 1°C and a relative humidity of 50 ± 5% throughout the preparation and testing of the samples. The materials from all three groups were mixed and prepared as previously described. After preparation, the materials were carefully packed into the molds using a mixing spatula, and dental pluggers were employed to ensure a uniform and dense fill with minimal porosity. The molds were then covered with glass slides at both ends to allow the materials to set completely ( Fig. 2 ). Once the set materials were removed from the molds, they were placed in an incubator at a temperature of 37°C and submerged in artificial saliva until the testing procedure was conducted. A total of 30 samples were made, with 10 samples per cement group. The compressive strength of the fully hydrated samples, which were submerged in artificial saliva, was measured using an Instron Universal Testing Machine (Hounsfield Test Equipment, Redhill, United Kingdom), as depicted in Fig. 3 .

A force was exerted along the long axis of the molds at a crosshead speed of 1 mm per minute until the materials were crushed. The peak force at which the set cement fractured was recorded in megapascals (MPa), representing the compressive strength of each sample. All 30 samples were tested individually, and the results were compiled for subsequent statistical analysis.

### Statistical Analysis

Data analysis was performed using IBM SPSS Statistics software version 22 (IBM Corp., Armonk, New York, United States). The Shapiro–Wilk test was utilized to evaluate the normality of the data distribution. One-way analysis of variance was used to analyze the compressive strength. For *post hoc* pairwise comparisons, Tukey's test was conducted. A *p* -value of less than 0.05 was regarded as statistically significant.

## Results

**Fig. 4:**
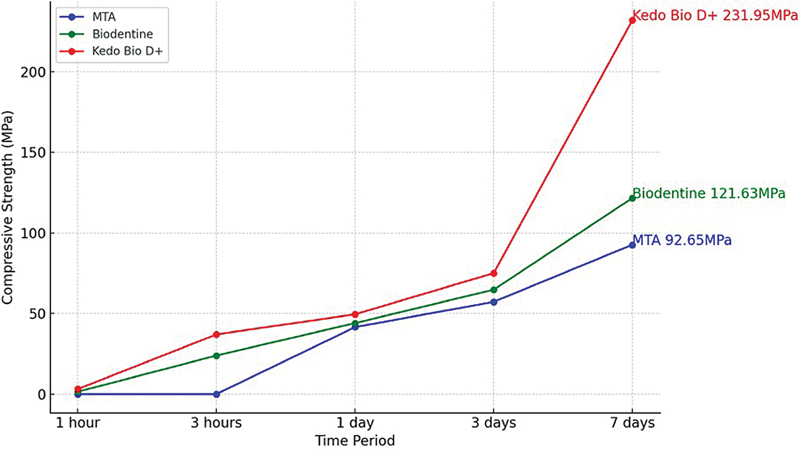
Graph 1: Comparison of the mean compressive strength of the different test materials at different time periods.

**Fig. 5:**
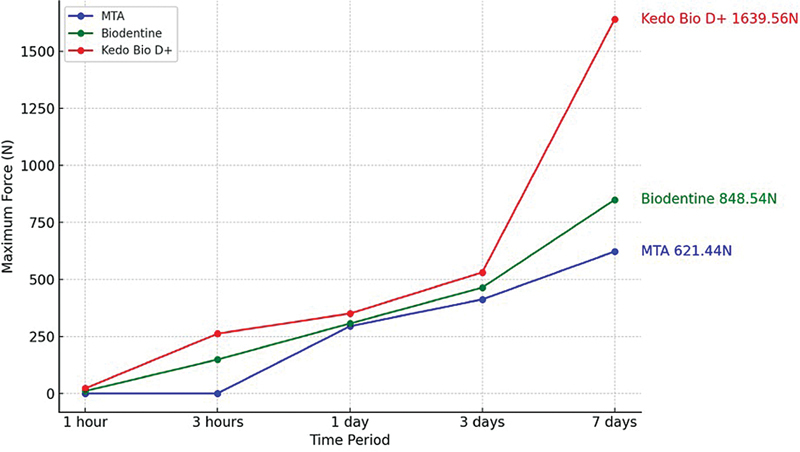
Graph 2: Comparison of the maximum force sustained by the different test materials at different time periods.

Table 1 shows the comparison of the mean compressive strength of the different test materials at different time periods of 1 hour, 3 hours, 1 day, and 3 days (Graph 1; Fig. 4 ). Among the tested materials, the highest mean compressive strength during all the time periods was seen in group 3, followed by group 2, and the least in group 1. This difference in compressive strength was statistically significant ( *p* < 0.05). Group C consistently demonstrated superior compressive strength and maximum force compared with group A and group B across all time intervals. This indicates that the modified formulation of biodentine in group C has enhanced mechanical properties, particularly its ability to withstand compressive forces, over time. A pairwise comparison shows statistically significant differences between all the groups included in the study ( *p* < 0.05) ( Table 2 ) (Graph 2; Fig. 5 ).

**Table 1:** Comparison of the maximum force sustained and mean compressive strength of the different test materials at different time periods

	Maximum force			*p* -Value	Compressive strength			*p* -Value
	Group A	Group B	Group C		Group A	Group B	Group C	
1 h	0	9.96	21.84	0.013	0	1.41	3.09	0.038
3 h	0	148.21	261.58	0.028	0	23.91	37.01	0.042
1 d	294.12	306.14	349.73	0.041	41.53	44.02	49.48	0.032
3 d	411.71	463.78	530.53	0.037	57.22	64.77	75.05	0.044
7 d	621.44	848.54	1639.56	0.001	92.65	121.63	231.95	0.001

*p* -Value<0.05.

**Table 2:** Pairwise comparison of different test materials

	Maximum force			Compressive strength		
	A-B	B-C	A-C	A-B	B-C	A-C
1 h	0.001*	0.021*	0.001*	0.001*	0.041*	0.001*
3 h	0.001*	0.036*	0.001*	0.001*	0.026*	0.001*
1 d	0.041*	0.051*	0.033*	0.045*	0.049*	0.037*
3 d	0.039*	0.038*	0.031*	0.042*	0.041*	0.038*
7 d	0.039*	0.001*	0.001*	0.031*	0.034*	0.001*

## Discussion

Over the past two centuries, advancements in material technology and treatment techniques have dramatically transformed endodontic practice. 17 Traditional materials and methods have been significantly modified in response to the growing demand for preserving and remineralizing tooth structure, alongside the development of novel cement technologies in recent decades. 18 Calcium hydroxide, once the standard material used in clinical practices for procedures such as indirect pulp capping and apexification, has had its limitations, prompting the need for materials with improved biological properties and clinical efficacy. 19 MTA, formulated by Torabinejad and Chivian, has proven to be a multifunctional and highly promising endodontic material. 20 It has successfully revived teeth with severe clinical conditions, providing hope for patients. MTA is widely used for pulp capping, pulpotomy, apexification, retrograde fillings, and perforation repairs. 2 However, MTA's tendency to discolor and its longer setting time have led to the development of newer bioceramics like biodentine, introduced over the last few years. 5 The evolving application of these cements has encouraged the incorporation of additives such as radiopacifiers, antimicrobial agents, alkalinizing agents, and calcium-releasing agents to improve their physical properties and bioactivity. 21 22

In pediatric dental practice, procedures such as pulp capping and apexification often require the use of fast-setting materials to facilitate final restoration within the same visit, which is especially crucial for young children and adolescents with short attention spans. 23 This study compared the new biodentine formulation with traditional materials, specifically conventional MTA and biodentine. MTA Angelus was chosen because of its excellent sealing capabilities, superior marginal adaptation, strong antibacterial effects, and adequate compressive strength. 24 Biodentine, manufactured by Septodont, was selected for its widespread use as a biomimetic material, attributed to its enhanced workability, quicker setting time, and higher compressive strength. 25 Both conventional MTA and biodentine are widely recognized as benchmarks in research involving Portland or tricalcium silicate-based cements. 26 Therefore, these materials were utilized as the reference groups for comparison. The present study aimed to reformulate biodentine to address the limitations of the original material, with a primary focus on enhancing its compressive strength.

Hydration accelerators have been suggested to improve the setting time, compressive strength, and overall quality of the material. 27 Tricalcium aluminate, in particular, has been proposed to aid in the faster binding of cement components, providing MTA-like consistency and setting properties. 28 29 30 However, concerns regarding the cytotoxicity of tricalcium aluminate have prompted the search for alternatives with better biological properties. 6 28 29 Compounds such as citric acid, lactic acid, calcium chloride, calcium lactate, disodium phosphate, polyethylene glycol, and calcium oxide have shown promise in enhancing cement setting properties and reducing cytotoxic effects. 28 31 In this study, calcium chloride at a 30% concentration was incorporated to accelerate the setting reaction of the new biodentine compared with conventional MTA and biodentine. The primary liquid component, polyethylene glycol, serves as a protective coating around tricalcium aluminate particles, limiting the release of alkaline ions and enhancing the material's biocompatibility. 32 33 34 The inclusion of polyethylene glycol as a protective layer is well-documented in previous studies. 32 33 34 This study primarily focuses on evaluating the compressive strength of the tricalcium silicate cements. Radiopacifiers such as zirconium oxide, barium oxide, calcium tungstate, and strontium carbonate are known to enhance compressive strength, while aluminate presence can reduce it. 35 Additionally, nanoparticles of zirconia and titanium have been proposed to improve compressive strength. 36 In this study, Kedo Bio D+ was tested wherein barium oxide, a radiopacifier, was added to the formulation; however, whether it made the new material more radiopaque than conventional biodentine or MTA was not assessed. Given the inclusion of multiple new components, *in vitro* testing was prioritized to examine the material's physical properties, with a particular focus on compressive strength, before advancing to *in vivo* or clinical trials.

The strength and durability of dental cements play a vital role in the success of restorations under clinical conditions. 37 Dental cements with higher compressive strength ensure dense packing within cavities, resulting in a secure seal and improved long-term outcomes. 37 Previous studies have demonstrated that biodentine exhibits superior compressive strength compared with MTA, attributed to the presence of calcium silicate hydrate colloidal gel in the set cement. 26 38 39 This observation aligns with findings in the literature, which reports favorable compressive strength for biodentine over MTA. 39 40 41 In the current study, Kedo Bio D+ showed significantly higher compressive strength than both conventional MTA and biodentine after 7 days. This enhancement can be credited to the addition of calcium chloride in the liquid component, which promotes stronger binding of the powder particles, thereby increasing the material's overall strength. 42

To facilitate a clearer understanding of the key differences between the three commercially available tricalcium silicate-based materials, a side-by-side comparison has been presented in Table 3 . This research article on the compressive strength of dental cements holds significant importance in pediatric dentistry. By understanding the mechanical properties of cements, pediatric dentists can select materials that can withstand masticatory forces, minimize microleakage, and indirectly support dentin bridge formation. This knowledge is critical in ensuring the long-term success of restorations in young patients, who are prone to higher rates of tooth decay and trauma.

**Table 3:** Comparison of key features among MTA, biodentine, and Kedo Bio D+

	Group A	Group B	Group C
Commercially available as	MTA Angelus	Biodentine Septodont	Biodentine Kedo Bio D+
Composition	Powder: Tricalcium silicate, dicalcium silicate, tricalcium aluminate, tetracalcium aluminoferrite, calcium sulfate, bismuth oxide, calcium oxide, silicon oxide, aluminum oxide Liquid: Distilled water	Powder: Tricalcium silicate, dicalcium silicate, calcium carbonate, iron oxide, zirconium oxide Liquid: Calcium chloride, hydrosoluble polymer, water	Powder: Tricalcium silicate, dicalcium silicate, calcium carbonate, tricalcium aluminate, calcium sulfate, barium oxide Liquid: Calcium chloride, polyethylene glycol
Mixing ratio	3:1 (powder: liquid)	5 drops of liquid for 1 capsule powder	3:1 (powder: liquid)
Setting time	Longer than biodentine	Manufacturer reported 12 minutes	Expected to be shorter due to calcium chloride addition
Compressive strength (after 7 days)	Lower than Kedo Bio D+ 57.22 MPa	Higher than MTA 64.77 MPa	Higher than MTA and biodentine 75.05 MPa

Abbreviation: MTA, mineral trioxide aggregate.

The limitations of this study include its *in vitro* design with a reasonable sample size. The results are specific to an *in vitro* setup and cannot be fully extrapolated to *in vivo* conditions without further analysis. The material's strength when exposed to salivary or blood contamination should also be assessed in future studies. Additionally, studies on cytotoxicity, push-out bond strength, mineralizing ability, *in* *vivo* clinical trials, calcium ion release, and long-term evaluation are yet to be done, which could further justify the clinical application of the newly formulated material. The pH of dental materials plays a critical role in their biocompatibility and clinical success. An alkaline pH is known to promote hard tissue formation and exhibit antibacterial properties, which are particularly advantageous in vital pulp therapies. In this study, the compressive strength of Kedo Bio D+ was significantly higher than that of conventional MTA and biodentine. However, the pH of the new biodentine was not evaluated. Future studies should prioritize the assessment of pH levels in Kedo Bio D+ to fully understand its biological effects. Furthermore, it would be beneficial to compare the pH of Kedo Bio D+ with that of conventional MTA and biodentine over time. This would provide valuable insights into the material's potential to promote pulp healing.

## Conclusion

Under the limitations of the present study, Kedo Bio D+ could serve as an alternative to the conventional tricalcium silicate cement, as it has a compressive strength that is significantly higher than conventional MTA and biodentine. The incorporation of barium oxide significantly enhanced its compressive strength. However, further cytotoxic and clinical studies are required to justify its clinical application.
